# Blockade of VLA4 sensitizes leukemic and myeloma tumor cells to CD3 redirection in the bone marrow microenvironment

**DOI:** 10.1038/s41408-020-0331-4

**Published:** 2020-06-01

**Authors:** Priyanka Nair-Gupta, Stephen I. Rudnick, Leopoldo Luistro, Melissa Smith, Ronan McDaid, Yingzhe Li, Kodandaram Pillarisetti, Jocelin Joseph, Bradley Heidrich, Kathryn Packman, Ricardo Attar, François Gaudet

**Affiliations:** Janssen Research & Development LLC, Spring House, PA USA

**Keywords:** Cancer microenvironment, Tumour immunology

## Abstract

Redirecting T cells to specifically kill malignant cells has been validated as an effective anti-cancer strategy in the clinic with the approval of blinatumomab for acute lymphoblastic leukemia. However, the immunosuppressive nature of the tumor microenvironment potentially poses a significant hurdle to T cell therapies. In hematological malignancies, the bone marrow (BM) niche is protective to leukemic stem cells and has minimized the efficacy of several anti-cancer drugs. In this study, we investigated the impact of the BM microenvironment on T cell redirection. Using bispecific antibodies targeting specific tumor antigens (CD123 and BCMA) and CD3, we observed that co-culture of acute myeloid leukemia or multiple myeloma cells with BM stromal cells protected tumor cells from bispecific antibody-T cell-mediated lysis in vitro and in vivo. Impaired CD3 redirection cytotoxicity was correlated with reduced T cell effector responses and cell–cell contact with stromal cells was implicated in reducing T cell activation and conferring protection of cancer cells. Finally, blocking the VLA4 adhesion pathway in combination with CD3 redirection reduced the stromal-mediated inhibition of cytotoxicity and T cell activation. Our results lend support to inhibiting VLA4 interactions along with administering CD3 redirection therapeutics as a novel combinatorial regimen for robust anti-cancer responses.

## Introduction

Despite several treatment options, there is currently no cure for acute myeloid leukemia (AML) and multiple myeloma (MM). Even after achieving high rates (50–80%) of complete hematologic remission (CR), defined as the presence of ≤5% of leukemic blasts (AML) or plasma cells (MM) in the bone marrow (BM)^1,2^, the majority of patients with AML or MM relapse^3–5^. Relapse has been linked to minimal residual disease (MRD) whereby small numbers of cancer stem cells (CSC), or other malignant progenitor cells, fail to be cleared and persist even after therapy^6^. Preventing relapses and finding cures for AML and MM requires finding better strategies to eliminate MRD.

Like hematopoietic stem cells (HSC), CSC in AML and MM reside and preferentially persist in the BM niche^7,8^. The BM niche provides a specialized microenvironment via secretion of soluble growth factors and cell–cell interactions that are protective to the CSC^9^. Moreover, the BM niche is immune-suppressive and is appreciated to be a site of immune privilege at steady state to allow for normal hematopoiesis and immune cell generation^10^. These aspects of the BM niche have provided resistance against and minimized the efficacy of several anti-cancer drugs including chemotherapy, targeted small molecule inhibitors, and antibody based therapies^11–14^.

The ability of T cells to specifically lyse tumor cells and secrete cytokines to recruit and support immunity against cancer makes them an attractive option for therapy. Several approaches have capitalized on this strategy such as bispecific T-cell engagers (BiTEs, small bispecific biologics), chimeric antigen receptors (CARs), and bispecific antibodies, among others^15^. BiTEs and antibody-mediated redirection cross-link T cells to tumor cells by engaging a specific epitope on tumor cells and CD3 on T cells, leading to T cell activation, and secretion of perforins and granzymes that ultimately kill the tumor cells. These CD3 redirection therapies have been validated as an effective anti-cancer strategy in the clinic with the approval of CD19xCD3 BiTE (blinatumomab) for acute lymphoblastic lymphoma (ALL)^16^. However, the immunosuppressive and protective nature of the BM niche potentially poses a significant hurdle to T cell redirecting therapies.

In this study, we investigated the impact of the bone marrow microenvironment on CD3 redirection. Using bispecific antibodies targeting specific tumor antigens (CD123 and BCMA) and CD3, we observed that co-culture of AML or MM cell lines with bone marrow stromal cells significantly protected cancer cells from bispecific-T-cell-mediated lysis in vitro. Similar results were observed in vivo when the presence of human bone marrow stromal cells in a humanized xenograft AML model attenuated tumor growth inhibition (TGI) observed with bispecific antibody treatment. Impaired CD3 redirection cytotoxicity was correlated with reduced T cell effector responses, thereby providing a mechanism to explain loss of activity of the bispecific antibody. Furthermore, our results indicate that cell-cell contact with stromal cells was crucial for reduced T cell activation and to confer protection of cancer cells. Finally, blocking the VLA4 adhesion pathway in combination with CD3 redirection abrogated the stromal-mediated inhibition of cytotoxicity and reversed stromal-mediated immunosuppression. Our results lend support to inhibiting VLA4 interactions along with administering CD3 redirection therapeutics as a novel combinatorial regimen for robust anti-cancer responses.

## Materials and methods

For detailed experimental procedures, please refer to the Supplemental methods.

### Antibody design

Antibodies were produced targeting human CD123/BCMA and CD3 and the lead antibody for CD123/BCMA and CD3 antibodies were joined together post-purification by generating a controlled fragment antigen binding arm exchange using the Genmab technology^17,18^. This resulted in a monovalent binding, bi-functional DuoBody® antibody which specifically binds to human CD123^+^ AML or human BCMA^+^ MM cells and CD3 T cells (Supplementary Fig. 1A and 1B). To minimize antibody-mediated effector functions, mutations were introduced in the Fc domain to reduce interactions with Fcγ receptors.

### In vitro and ex vivo cytotoxicity assays

Tumor cell lines were labeled with CFSE and co-cultured with thawed purified frozen T cells in the presence or absence of stromal cell lines (HS-5 and HS-27a), primary mesenchymal stromal cells (MSC) and CD105^+^ endothelial cells. In all, 24 h later, bispecific antibodies were added to the wells and the plates were incubated at 37 °C with 5% CO_2_ for 48 h. The cells were then stained for various markers before analyzing on the flow cytometers. For the trans-well related experiments, the assay was performed in 96-well U bottom plates with or without 0.4 µm transwell inserts (HTS TRANSWL96, Corning). For the IncuCyte^®^ related experiments, red fluorescent OCI-AML5 cells (OCI-AML5-NucLight Red) and green HS-5 (HS-5-NucLight Green) were used.

For the ex vivo assays, HS-5 cells were plated prior to addition of AML PBMCs or MM BMMCs. CD123xCD3 or BCMAxCD3 or nullxCD3 bispecific antibodies (1 µg/ml) with or without anti-VLA4 antibody (5 µg/ml) were added. In all, 72 h later, depletion of CD123^+^ blasts or CD138^+^ MM plasma cells was monitored via flow cytometry. Additionally, expansion of CD8 T cells as well as their activation status (upregulation of CD25) were assessed.

### In vivo MOLM-13 xenograft model

Human PBMC (1 × 10^7^ cells/mouse) were inoculated intravenously (iv) 6–7 days prior to tumor cell implantation. On study day 0 mice were implanted subcutaneously (sc) with 1 × 10^6^ MOLM-13 cells and two concentrations of HS-5 bone marrow stromal cells, 2 × 10^5^ and 5 × 10^5^. Treatments with CD123 x CD3 (0.04 mg/kg and 0.008 mg/kg, *n* = 8) or vehicle PBS controls (*n* = 5) were given intravenously (iv) every 3 days (*q3d*) for 5 doses. Individual mice were monitored for body weight loss and tumor growth inhibition twice weekly for the duration of the study. In the case of the in vivo study with the VLA4 blocking antibody, treatments with CD123 × CD3 bispecific antibody (0.008 mg/kg, *n* = 8 or 9) or PBS vehicle control (*n* = 5) were given iv and the anti-VLA4 antibody (5 mg/kg) given intraperitoneally (ip). No animals were excluded from the analysis.

### Statistical methods

**Fig. 1:**
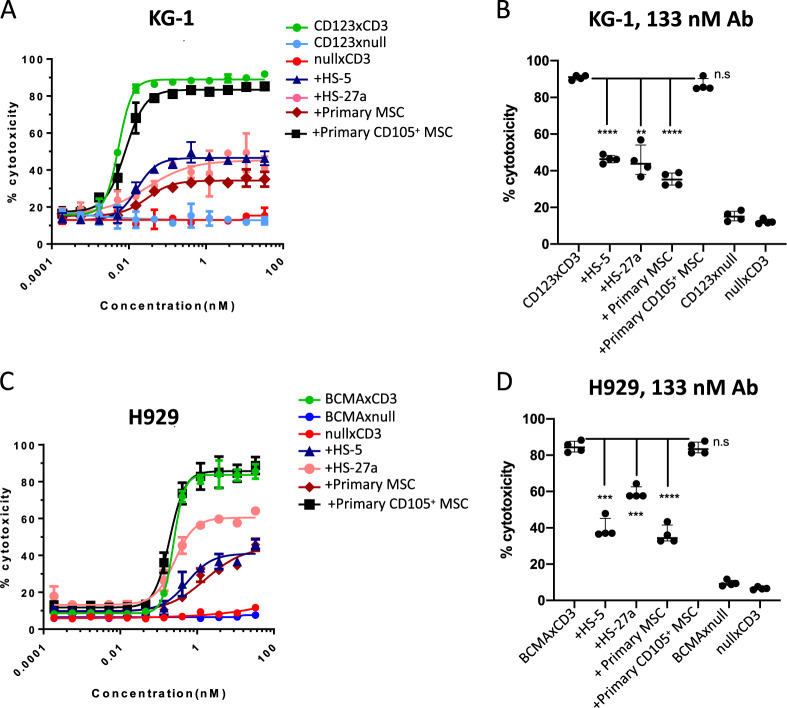
Presence of stromal cells protects AML and MM cell lines from T cell redirected cytotoxicity. Human T cells (40,000 cells/well) were cultured with CFSE labeled AML KG-1 (**a**, **b**) or MM H929 cell lines (**c**, **d**) 2:1 ratio in the presence or absence of stromal cells (HS-5, HS-27a, primary MSC, or CD105^+^ endothelial cells; 20,000 cells per well). Varying concentrations of CD123xCD3 (**a**, **b**) or BCMAxCD3 (**c**, **d**) were added to cultures for 48 h. The percentage of dead CFSE^+^ cells was quantitated by flow cytometry. Dose titration graphs on the left (**a**, **c**) are shown with means ± standard deviation (SD). Scatter plots on the right (**b**, **d**) show data for the highest concentration of the bispecific antibody (median with range). Data are representative of three or more experiments. ∗∗*p* < 0.005, ∗∗∗*p* < 0.0005; ∗∗∗∗*p* < 0.0001; n.s. not statistically significant.

**Fig. 2:**
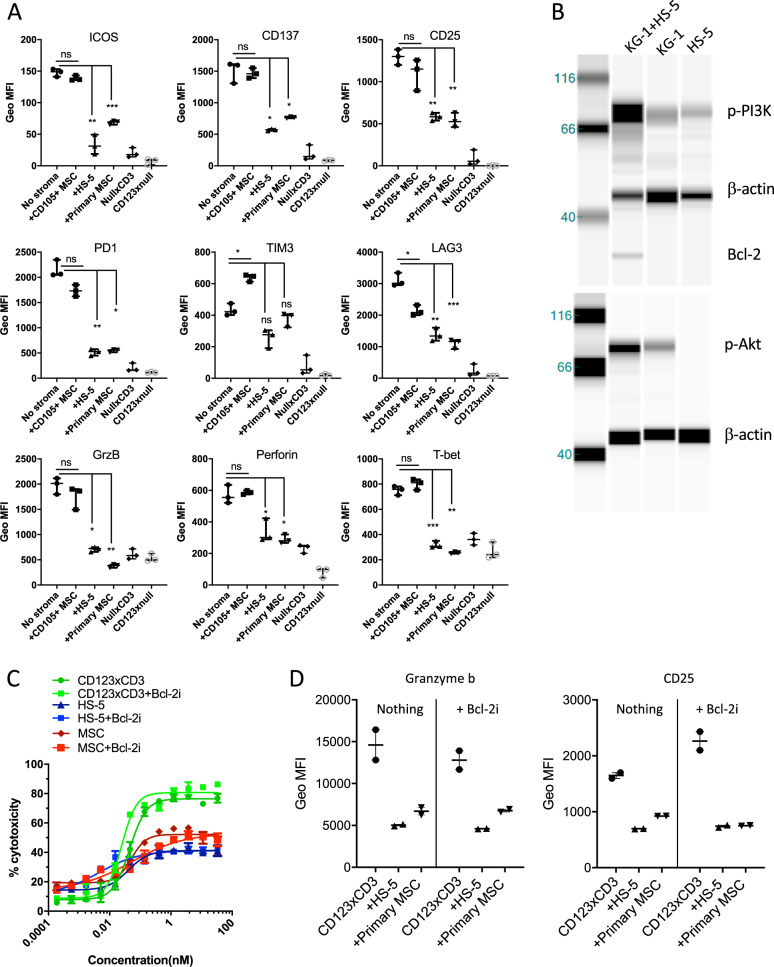
Stromal cells suppress T cell function, upregulate signaling pathways in tumor cells and protect tumor cells from T cell redirected cytotoxicity. **a** Human T cells (40,000 cells/well) were cultured with CFSE labeled KG-1 cells at a 2:1 ratio in the presence or absence of stromal cells (HS-5, primary MSC or CD105^+^ endothelial cells; 20,000 cells per well). Analysis of activation, effector, and checkpoint inhibition markers in CD8^+^ T cells was performed post addition of 33 nM of CD123xCD3 to cultures. Geometric mean fluorescence intensities were quantified by flow cytometry at 48 h. **b** Immunoblotting analysis of activation (phosphorylation) of PI3K and Akt as well as expression of Bcl-2 in KG-1 cells cultured either alone or in the presence of HS-5 stromal cell line for 48 h. **c** Similar to **a** but here all the cultures were treated with or without Bcl-2i and the percentage of dead CFSE^+^ cells was quantitated by flow cytometry. **d** Similar to **a** and **c** where activation status of T cells was assessed in tumor and T cell cultures in the absence or presence of stroma and with or without treatment with Bcl-2i. All data shown are representative of three or more experiments and are depicted as either mean with SD (dose titration curves) or median with range (scatter plots). ∗*p* < 0.05, ∗∗*p* < 0.005, ∗∗∗*p* < 0.0005; ∗∗∗∗*p* < 0.0001; n.s. not statistically significant.

**Fig. 3:**
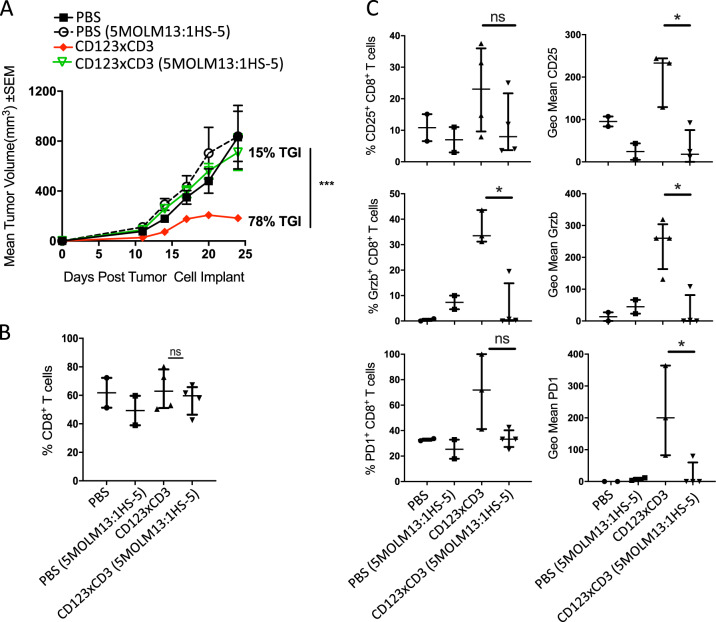
Stromal cells impact efficacy of CD3 redirection in vivo. MOLM-13 AML and MOLM-13 with HS-5 bone marrow stromal cells (5:1) were implanted sc in huPBMC injected female NSG mice on study day 0. Mice were treated with CD123xCD3 (8 μg/kg) starting on day 5 post tumor cell implant twice weekly for a total of 5 treatments. PBS-treated groups were included as controls. **a** Mean tumor volume measurements for all the groups at different time points. **b** Percentage of CD8^+^ T cell infiltration in the tumors of mice at the end of the study (day 24). **c** Analysis of activation, effector, and checkpoint inhibition markers in CD8^+^ T cells in the tumors of mice on day 24. All data shown are representative of two independent experiments and are represented as either mean ± standard error of mean (SEM) (**a**) or median with range (**b**, **c**). ∗*p* < 0.05, ∗∗*p* < 0.005, ∗∗∗*p* < 0.0005; n.s. not statistically significant.

**Fig. 4:**
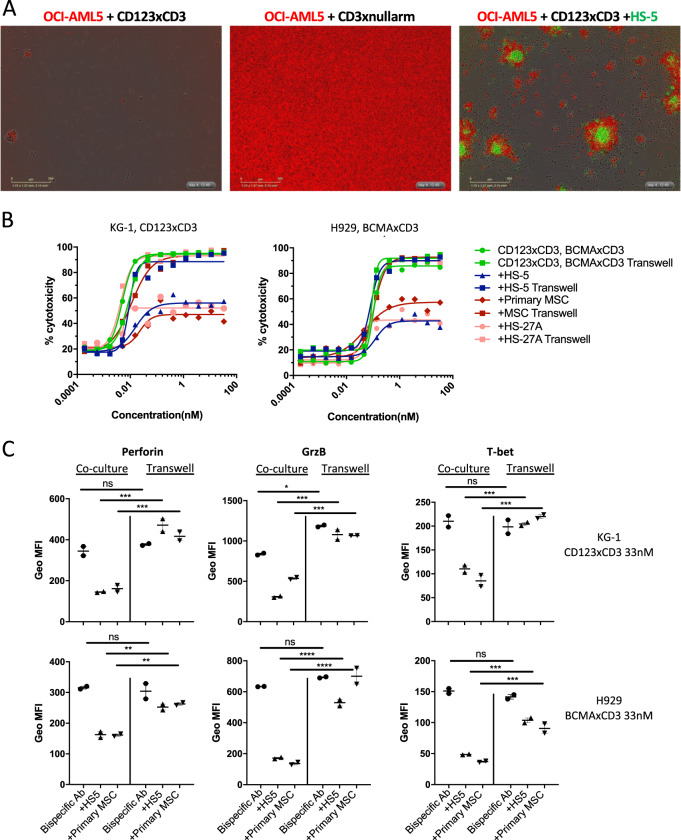
Cell–cell contact plays a dominant role in mediating the immune-suppressive and protective phenotype of stromal cells. **a** Human T cells (40,000 cells/well) were cultured with Incucyte NucLight® Red labeled OCI-AML5 cells (20,000) with or without Incucyte NucLight® Green labeled HS-5 cells (20,000 cells per well) and were treated with varying concentrations of bispecific antibody for 72 h. Representative images show a snapshot of the cultures at 72 h post addition of 11 nM of CD123xCD3 bispecific antibody. **b** Same as A but here the T cells were cultured with CFSE labeled tumor cells with or without stromal cells (HS-5 or primary MSC) and were treated with varying concentrations of bispecific antibody for 48 h. In these assays, stromal cells were either cultured together or separated from the tumor and T cells in a trans-well. The percentage of dead CFSE^+^ cells was quantitated by flow cytometry. Data from one experiment shown here which is representative for three independent biological repeats. Data shown here as mean ± SD. **c** Flow analysis of activation and effector markers on CD8^+^ T cells in the killing assays. Data shown as median with range. ∗*p* < 0.05, ∗∗*p* < 0.005, ∗∗∗*p* < 0.0005; n.s. not statistically significant.

**Fig. 5:**
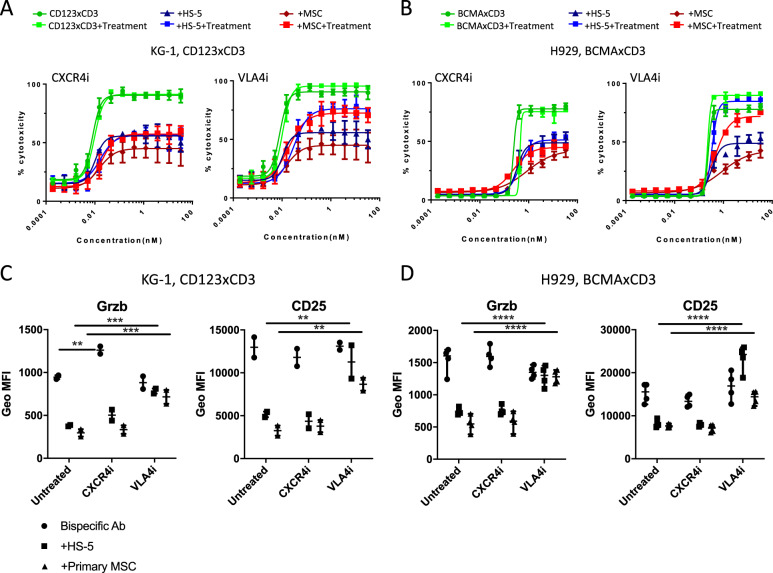
VLA4 inhibition reverses stromal-mediated immune-suppression and protection of tumor cells from CD3 redirected cytotoxicity in vitro. Human T cells were cultured with CFSE labeled tumor cells with or without stromal cells (HS-5 or primary MSC) and were treated with varying concentrations of bispecific antibody for 48 h in the presence or absence of neutralizing antibodies to VLA4 or CXCR4. **a**, **b** The percentage of dead CFSE^+^ cells was quantitated by flow cytometry. **c**, **d** Flow analysis of granzyme B and CD25 expression on CD8^+^ T cells in the killing assays. Data are representative of three or more experiments and are represented as mean ± SD (**a**, **b**) and median with range (**c**, **d**). ∗*p* < 0.05, ∗∗*p* < 0.005, ∗∗∗*p* < 0.0005; ∗∗∗∗*p* < 0.0001; n.s. not statistically significant.

**Fig. 6:**
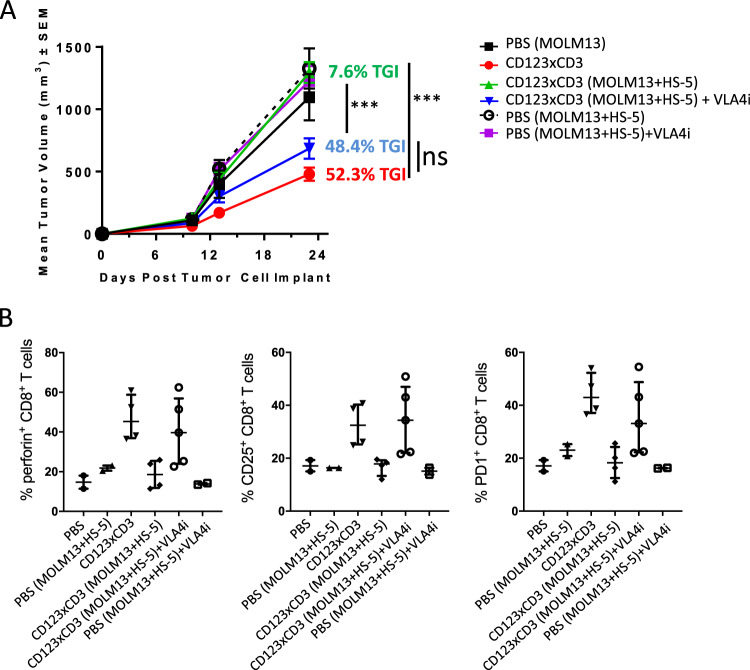
VLA4 inhibition reverses stromal-mediated immune-suppression and protection of tumor cells from CD3 redirected cytotoxicity in vivo. AML cell line MOLM-13 and MOLM-13 with HS-5 bone marrow stromal cells (5:1) were implanted sc in huPBMC injected female NSG mice on study day 0. Mice were treated with CD123xCD3 (8 μg/kg) either alone or in combination with a neutralizing antibody against VLA4 (3 mg/kg). PBS-treated groups were included as controls. **a** Mean tumor volume measurements for all the groups at different time points. **b** Analysis of activation, effector, and checkpoint inhibition markers in CD8^+^ T cells in the tumors of mice on day 23. All data shown are representative of two independent experiments and are represented as mean ± SEM (**a**) and median with range (**b**). ∗*p* < 0.05, ∗∗*p* < 0.005, ∗∗∗*p* < 0.0005; ∗∗∗∗*p* < 0.0001; n.s. not statistically significant.

**Fig. 7:**
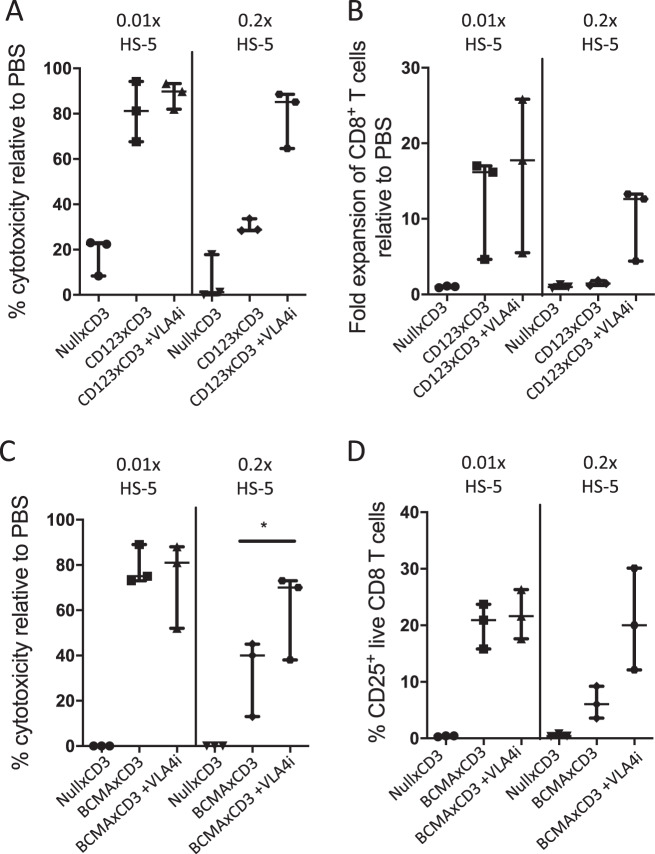
VLA4 inhibition rescues efficacy of CD3 redirection in ex vivo primary AML and MM cultures. PBMCs from three primary AML samples (**a**, **b**) or BMMCs from three MM samples (**c**, **d**) were incubated with bispecific antibodies at 1 μg/mL in the presence of HS-5 and with/without neutralizing antibodies against VLA4 for 72 h. Median values with range are depicted for cytotoxicity (**a**, **c**) or CD8 T cell expansion (**b**)/activation (**d**) for all three primary samples. ∗*p* < 0.05.

Data were analyzed by GraphPad software Prism version 8 (SAS Institutes, Cary, NC). Browne-Forsythe and Welch ANOVA test analysis was applied for Figs. 1 and 2 while ordinary 2-way ANOVA analysis was applied to Figs. 3–7.

## Results

### BM stromal cells protect AML and MM cell lines from CD3 bispecific antibodies and T cell-mediated cytotoxicity

The BM niche is characterized by its protective and immune-suppressive microenvironment. We used BM stromal cells to mimic the BM niche as they are a major cellular component of the endosteal and vascular niches that govern fundamental HSC cell fate decisions including self-renewal, survival, differentiation, and proliferation^19,20^. BM stromal cells are also documented to mediate immune-suppression^13,21^ while also activating multiple survival and anti-apoptotic pathways in tumor cells, thus allowing them to become resistant to different types of therapy^22^. We thus co-cultured AML or MM cell lines, T cells and bispecific antibodies in the absence or presence of BM stromal cells. We used bispecific antibodies targeting either CD123 or BCMA and CD3 (tool antibodies). Binding, killing, and T cell activation data demonstrating efficacy of these antibodies are shown in Supplementary Fig. 1. Using the CD123xCD3 bispecific antibody, we observed dose-dependent killing of CD123-expressing leukemic cell line KG-1 (Fig. 1a, b). This killing was not observed with bispecific antibodies that express either CD3 or CD123 along with a non-targeting (null) arm (Fig. 1a, b). Similar results were observed when using BCMA expressing MM cell line H929 and another bispecific antibody BCMAxCD3 (Fig. 1c, d) where specific killing was mediated by BCMAxCD3 in contrast to the null controls. When stromal cells were added to the co-culture, we observed a statistically significant decrease in the maximum observed cytotoxic response, even at high concentrations of the bispecific antibodies (Fig. 1a–d). Furthermore, EC_50_ values of the bispecific antibody were 3–5-fold higher in the presence of stroma (Supplementary Fig. 2A). Stromal inhibition of bispecific antibody activity was not merely restricted to fibroblast stromal cell lines (HS-5 and HS-27a) but was also observed with primary mesenchymal stromal cells (MSC) derived from the BM of healthy donors (Fig. 1a–d). Inhibition of bispecific antibody activity was dependent on the number of stromal cells present in the co-culture; whereby decreased efficacy was still observed when stromal cells were 10 -fold less than cancer cells in the co-cultures (Supplementary Fig. 2B). Interestingly, we did not observe any inhibition with the addition of CD105^+^ endothelial cells sorted from BM mononuclear cells of healthy donors (Fig. 1a–d). This result demonstrated that not all stromal cells adversely impacted the efficacy of CD3 redirection bispecific antibodies and also accounted for the fact that the mere presence of another cell type in the tumor-T cell co-culture did not contribute to the inhibition effect. Stromal-mediated inhibition of bispecific antibody activity was not unique to T cells from one donor but was observed with T cells of multiple donors (means and medians with different donors plotted in Figs. 1a, c and 1b, d respectively). These data were not specific to one AML or MM cell line as similar results were observed with other CD123^+^ AML (MOLM-13 and OCI-AML5) and BCMA^+^ MM cell lines (RPMI-8226 and MM.1S) (Supplementary Fig. 2C, D). These data demonstrate for the first time that stromal cells can impact the efficacy and potency of CD3 redirection bispecific antibodies.

### BM stromal cells suppress T cell activity and activate survival and anti-apoptotic pathways in cancer cells

We next investigated the mechanisms underlying stromal inhibition of bispecific antibody activity. To this end, we assessed the phenotype of T cells in our T cell-tumor co-culture cytotoxicity assays in the absence or presence of stromal cells. Treatment with CD123xCD3 bispecific antibody in the absence of stroma resulted in the upregulation of activation markers including CD25, CD137 and ICOS with concomitant increases in checkpoint markers including PD1, LAG3 and TIM3 in CD8^+^ T cells (Fig. 2a). Additionally, the CD8^+^ T cells exhibited characteristics of cytotoxic T lymphocytes (CTL) by increased production of effector proteins such as perforin and granzyme B and upregulation of T-bet expression (Fig. 2a). However, when stromal cells were present in the co-culture, T cells were less activated with reduced expression of activation, effector and checkpoint markers (Fig. 2a). These results were observed with multiple T cell donors (medians with different donors plotted in Fig. 2a) as well as with MM cell line H929 and the BCMAxCD3 bispecific antibody (Supplementary Fig. 3A). The results with the decreased expression of checkpoint markers on T cells in the presence of stroma may seem counterintuitive at first given that PD1, TIM3, and LAG3 are recognized as inhibitory proteins that regulate T cell activation response. However, these proteins are induced only upon T cell activation and are absent in naive T cells^23–29^. Given these data, it is therefore not surprising that the upregulation of PD1, TIM3 and LAG3 is diminished on T cells in the presence of stromal cells and support the less activated phenotype of the T cells in the presence of the inhibitory stromal compartment. Furthermore, T cell proliferation was reduced in the presence of stroma, post-treatment with both bispecific antibodies (Supplementary Fig. 3B).

In addition to immune suppression, we investigated if stromal-mediated activation of multiple pro-survival and anti-apoptotic pathways in leukemic and myeloma tumor cells could be an additional mechanism to mediate resistance against therapy^30^. We observed increased phosphorylation of phosphoinositide 3-kinase (PI3K) and Akt and increased protein expression of Bcl-2 in KG-1 cells that were cultured with HS-5 stromal cells and not in KG-1 or HS-5 cells alone (Fig. 2b). Together these data suggest that AML and MM tumor cells can evade T cell-mediated death by a stromal cell-dependent mechanism involving activation of resistance pathways in tumor cells in addition to suppressed activation of T cells.

We next investigated the relative contribution of T cell immune suppression and upregulation of pro-survival pathways to the phenotype of reduced efficacy of CD3 redirection. Given that Bcl-2 has been directly implicated in survival and resistance of AML and MM cells from several therapies^30,31^, we performed cytotoxicity assays in the presence of stroma with or without the addition of a Bcl-2 inhibitor HA14-1. While the inhibitor successfully prevented expression of Bcl-2 (Supplementary Fig. 4), it did not rescue stromal-mediated inhibition of CD3 redirection (Fig. 2c) and T cells remained less activated (Fig. 2d). These data support previously published findings where overexpression of Bcl-2 in target cells had minimal impact on the activity of AMG110 (EpCAMxCD3 BiTE)^32^. We thus focused on suppression of T cell activation and function as the mechanism to explain stromal-mediated inhibition of CD3 redirection.

### Bone marrow stromal cells attenuate efficacy of CD3 redirection in vivo

We next investigated if stromal cells could protect tumor cells from bispecific antibodies-T cell-mediated cytotoxicity in vivo. To this end, human peripheral blood mononuclear cells (PBMCs) were intravenously inoculated in female NSG mice and one week later, MOLM-13 with or without HS-5 bone marrow stromal cells were implanted subcutaneously (sc) on the flank of the mice. Mice were then treated with CD123xCD3 (8 μg/kg) starting on day 5 post tumor cell implant twice weekly for a total of 5 treatments. Treatment with CD123xCD3 significantly inhibited sc tumor growth (tumor growth inhibition (TGI _Day 25_) = 78%, *p* < 0.0001) in the MOLM-13 alone group compared to PBS or CD3xnull controls (Fig. 3a). This anti-tumor activity was markedly reduced (TGI_Day 25_ = 15%) in the presence of stroma and was statistically significant compared to bispecific antibody treated MOLM-13 alone group (*p* < 0.0001) (Fig. 3a). Furthermore, while we observed equal infiltration of CD8^+^ T cells in tumors with or without stroma (Fig. 3b), we noted differences in the activation profiles of the T cells that correlated to the presence of stroma (Fig. 3c). CD8^+^ T cells from bispecific antibody treated MOLM-13+HS-5 groups exhibited impaired upregulation of CD25, PD1 and granzyme B compared to the MOLM-13 controls (Fig. 3c). These results support our in vitro observations and strongly suggest that BM stromal cells reduce the efficacy of otherwise potent CD3 redirection therapeutics by suppressing T cell activation.

### Adhesion to stroma is critical to mediate immune suppression and cancer cell survival

Stromal cells can mediate immune-suppression and protect tumor cells from cytotoxicity via secretion of soluble factors including immune suppressive mediators such as IL-10, TGF-β and PGE2 or growth factors such as stem cell factor (SCF), IL-7, IL-15, CXCL-12 among others^21,33^. Additionally, stromal cells can directly interact with tumor cells via adhesion pathways inducing resistance^34^ and thereby protect malignant cells from T cell-mediated cytotoxicity in a cell-cell contact dependent manner. Visual examination of the cytotoxicity assays revealed that residual leukemic cells not killed by bispecific antibody-T-cell-mediated cytotoxicity clustered closely around stromal cells (Fig. 4a), suggesting that cell–cell contact pathways may play an important role in stromal-mediated protection of cancer cells. To discern between soluble vs cell–cell contact dependent mechanisms, we performed in vitro transwell assays to assess if stromal cells could still inhibit efficacy of bispecific antibody-T-cell-mediated lysis even if separated from tumor and T cells. We observed that cell–cell contact played a dominant role in mediating the stromal protection of AML and MM cell lines from bispecific antibody-T-cell redirected cytotoxicity since the stromal cells did not exhibit any inhibitory effect when separated from the tumor cells (Fig. 4b and Supplementary Fig. 5A). Similar trends were observed with T cells from different donors (two different T cell donors used in Fig. 4b). Moreover, when stromal cells were placed in a transwell insert, they were unable to suppress T cell activation and expression of perforin, granzyme B and T-bet (Fig. 4c). These data demonstrate the strong dependence on cell-cell interactions for stromal-mediated T cell suppression and protection from T-cell-dependent cytotoxicity.

### Blocking VLA4 in vitro and in vivo rescues stromal-mediated inhibition of CD3 redirection

We next investigated which of the adhesion pathways was critical for stromal inhibition of bispecific antibody efficacy. We focused on CXCR4 and VLA4 because of their documented roles in mediating AML/MM-stroma interactions in the BM^34^. Using blocking antibodies against either VLA4 or CXCR4, we observed that unlike CXCR4 inhibition which failed to rescue bispecific antibody-mediated cytotoxicity responses in the presence of stroma, VLA4 inhibition reversed (50–60%) stromal-mediated protection of KG-1 and MOLM-13 from CD123xCD3 bispecific antibody-T cell cytotoxicity (Fig. 5a and Supplementary Fig. 5B). This effect was more pronounced with H929 and BCMAxCD3 bispecific antibody where VLA4 inhibition restored cytotoxicity responses (80–100%) even in the presence of stroma (Fig. 5b). Increased cytotoxic responses with VLA4 inhibition correlated with restored expression of T cell activation markers such as granzyme B and CD25 which were still repressed under the CXCR4 blockade (Fig. 5c, d). This increase in T cell activation markers with VLA4 inhibition was also statistically significant compared to the untreated counterparts (co-cultures containing either HS-5 or primary MSC stromal cells). VLA4 inhibition also attenuated the phosphorylation of Akt and PI3K pathways (Supplementary Fig. 6).

Our previous in vivo results had shown that the efficacy of CD123xCD3 was attenuated in treating MOLM-13 tumors with HS-5 bone marrow stromal cells. To determine if the anti-tumor effect can be restored, an anti-VLA4 neutralizing antibody was combined with CD123xCD3 for the treatment of MOLM-13-bearing mice. Similar to our prior observations, CD123xCD3 (8 µg/kg) promoted a TGI_Day 24_ of 52.3% (*p* ≤ 0.0001) compared to PBS-treated controls while the same dose of bispecific antibody had minimal effects against MOLM-13 tumors co-injected with HS-5 cells (TGI_Day 23_ = 7.6%) (Fig. 6a). However, the concomitant addition of anti-VLA4 antibody with CD123xCD3 resulted in increased TGI_Day 23_ of MOLM-13 tumors with HS-5 cells of 48.4%, (*p* = 0.0001) (Fig. 6a). Increased TGI of MOLM-13 tumors with stroma receiving VLA4 blockade and bispecific antibody treatment was accompanied by improved CD8^+^ T cell activation and effector responses including expression of perforin, CD25 and PD1 (Fig. 6b). The increased TGI and augmented CD8^+^ T cell response was limited to those tumor+HS-5 bearing mice receiving the combination of VLA4 blockade and bispecific antibody treatment and was absent when the mice were dosed with either agent by themselves. These results strongly suggest that concomitant blockade of VLA4 along with CD3 redirection agents can overcome the suppressive effects mediated by stromal cells and can mediate superior anti-tumor responses.

### Blocking VLA4 in ex vivo primary patient cultures restores efficacy of CD3 redirection despite the presence of stroma

We then verified our findings with primary frozen/thawed AML and MM samples. Given that primary tumor cells can be a challenge to maintain in culture without exogenous supplementation of cytokines or stromal support, we performed ex vivo cultures of AML/MM samples with varying numbers of stromal cells (representative gating strategy shown in Supplementary Figs. 7 and 8). Strikingly, we observed that there was appreciable and selective killing of CD123^+^ CD33^+^ AML blasts as well as BCMA^+^ CD138^+^ MM tumor cells in CD123xCD3 and BCMAxCD3 bispecific antibody treated cultures that had low stroma: tumor ratio (0.01x HS-5) and not when the cultures were treated with null controls (Fig. 7a, c). This killing effect mediated by both bispecific antibodies was reduced in cultures with high stroma: tumor ratio (0.2x HS-5, Fig. 7a, c). Additionally, effective cytotoxic responses of clearing primary tumor cells were followed by expansion or activation of CD8^+^ T cells that were restricted to bispecific antibody treated cultures that had less stroma content (Fig. 7b, d). Lastly, when we neutralized VLA4 in combination with CD123xCD3 or BCMAxCD3, we observed superior killing of tumor cells and restoration of efficacy of bispecific antibody despite the higher stromal content in the cultures (Fig. 7a–d). Blocking VLA4 along with the bispecific antibody treatment also restored expansion/activation of CD8^+^ T cells in the cultures that had a higher stromal content (Fig. 7b, c). These results were observed with three different patient samples (AML patients in Fig. 7a, b and MM patients in Fig. 7c, d) and strengthen our previous in vitro and in vivo findings. VLA4 inhibition by itself resulted in the increased depletion of CD123^+^ blasts in the cultures with high stroma: tumor ratio in one of the AML patient samples but this effect was not broadly observed (data not shown). These data indeed demonstrate that combining VLA4 blockade with CD3 redirection bispecific antibody therapeutics can overcome suppressive effects of stromal cells and provide rationale for exploring such combinations in the clinic.

## Discussion

The complexity of the bone marrow niche has truly been appreciated in the recent years with significant advancements in understanding the molecular and cellular factors that contribute toward maintenance and regulation of hematopoietic stem cells. In the context of hematological malignancies, the same factors can be exploited by cancer stem cells for protection from and resistance to several anti-cancer therapies, thus contributing to minimal residual disease.

Our results show for the first time how otherwise effective T cell therapeutics can be thwarted by components of the BM microenvironment. Specifically, we observed that in the presence of BM stromal cells, AML and MM cancer cells were protected from cytotoxicity mediated by T cell and bispecific antibodies. Reduced killing of cancer cells correlated with blunted T cell activation and effector responses. Blocking cell–cell interactions specifically those mediated by the VLA4 pathway reversed T cell immune suppression leading to increased killing of AML and MM cancer cells. Our results thus reaffirm that the bone marrow microenvironment is a formidable factor that needs to be considered even in the context of otherwise potent and effective immune therapies such as CD3 redirection. Our results also provide rationale and evidence for combining agents that interfere with adhesion with CD3 redirection therapeutics for better and more complete elimination of MRD.

While we demonstrate that blocking VLA4 reverses stromal inhibition of efficacy of bispecific T cell-mediated cytotoxicity and immunosuppression, the mechanisms underlying this regulation remain to be delineated. VLA4 is expressed on T cells and can provide costimulatory signals resulting in activation of T lymphocytes in addition to mediating adhesion and transendothelial migration of leukocytes^35–38^. Clinical studies in multiple sclerosis patients with natalizumab, a humanized monoclonal IgG_4_ VLA4 blocking antibody approved for MS, have shown that the drug not only increases the number of CD4^+^ and CD8^+^ T cells in the peripheral blood^39^ but also stimulates CD4^+^ and CD8^+^ T cell production of more IL-2, TNF-α, IFN-γ, and IL-17^40–43^. While the results were more modest, similar results were observed in vitro where natalizumab induced a mild upregulation of IL-2, IFN-γ, and IL-17 expression in activated primary human CD4^+^ T cells propagated ex vivo from healthy donors, suggesting that natalizumab directly acts on T cells^42^. The above study was focused on CD4^+^ T cells; so whether the same is observed for CD8^+^ T cells in vitro remains to be investigated. Another mechanism to explain the effect of VLA4 blockade could be that blocking interaction between tumor and stroma cells disrupts clustering of tumor cells around the stroma, thus allowing the T cells to access the tumor cells, leading to better efficacy of CD3 redirection. Lastly, VLA4 inhibition has been shown to directly act on AML and MM cells, rendering them more susceptible to chemotherapy and targeted therapies by preventing the expression and upregulation of key pro-survival pathways in the tumor cells themselves^34^ or altering tumor cell production of anti-inflammatory cytokines.

While this study was focused on the bone marrow microenvironment, it is possible that a similar phenomenon occurs in solid tumors. Solid tumors contain a complex dense network of extracellular matrix molecules as well as a variety of stromal cell types that may be immunosuppressive. Indeed, CD3-redirection strategies have yet to show clinical benefits in solid tumor malignancies^44,45^ and inhibition by stromal cells and factors may be a contributing factor. It would be interesting to test the efficacy of CD3 redirection therapeutics in the presence of epithelial associated stroma such as cancer associated fibroblasts and test if interfering with adhesion pathways similarly rescues efficacy. Indeed, CXCL12 production by stromal cells has been reported to contribute to T cell exclusion in murine models of pancreatic ductal carcinoma^46^. In these models, CXCR4 inhibition resulted in rapid T cell infiltration and synergized with PD-L1 immunotherapy leading to better clearance of the tumor cells^46^. Whether VLA4 inhibition would lead to a similar effect and in the context of combination with CD3 redirection therapies remains to be investigated in solid tumors.

Furthermore, while this study focused on using a limited panel of stromal cell types such as fibroblasts, endothelial cells and mesenchymal stromal cells, it is important to also understand the contribution of other types of stromal cell types include osteoblasts, chondrocytes, adipocytes and CXCL12-abundant reticular (CAR) cells within the bone marrow microenvironment towards resistance of CD3 redirection. Additionally, it would be interesting to test and compare stromal cells derived from normal healthy donors to those derived from AML and MM patients. Moreover, we anticipate that other CD3 redirecting bispecific antibodies generated via alternative platforms or other bispecific biologic scaffolds to be impacted similarly by the bone marrow microenvironment.

Our results point toward the importance of targeting the bone marrow microenvironment in conjunction with CD3 redirection therapies. Additionally, our results suggest that VLA4 could potentially be used as a biomarker to predict responses toward CD3 redirection and perhaps used to guide patient selection for these immune therapies. Factoring in the bone marrow microenvironment to guide the design of not only novel combinatorial regimens but also better clinical trials has the potential to have a major impact in the successful elimination of MRD and the achievement of cures in hematological malignancies.

## Supplementary information

Supplementary Figure legends

Supplementary Methods

Supplemental Figure 1

Supplemental Figure 2

Supplemental Figure 3

Supplemental Figure 4

Supplemental Figure 5

Supplemental Figure 6

Supplemental Figure 7

Supplemental Figure 8
