# Supplementary material for: Blockade of VLA4 sensitizes leukemic and myeloma tumor cells to CD3 redirection in the bone marrow microenvironment
Source: Blood Cancer J. 2020 Jun 1;10(6):65. doi: 10.1038/s41408-020-0331-4 (PMC7264144; doi:10.1038/s41408-020-0331-4)
Supplement: Supplementary file 1 — Supplementary Figure legends [file 41408_2020_331_MOESM1_ESM.docx]

**Supplementary Figure legends**

**Figure 1. CD123xCD3 and BCMAxCD3 bind tumor cells as well as mediate killing and T cell activation**

(A) CD123^+^ or BCMA^+^ cell lines were stained with various concentrations of the bispecifc antibodies to characterize the surface binding profiles. Binding of the bispecific antibody was detected by staining with mouse anti-human IgG_4_. (B) Ability of CD123xCD3 and BCMAxCD3 to mediate T cell activation (measured by CD25 upregulation and production of granzyme b) and cytotoxicity of CD123^+^ or BCMA^+^ tumor cell lines.

**Figure 2. Presence of stromal cells protects AML and MM cell lines from T cell redirected cytotoxicity**

Human T cells were cultured with CFSE labelled AML or MM cell lines at a 2:1 ratio in the presence or absence of stromal cells (HS-5, HS-27a, primary MSC or CD105^+^ endothelial cells). Varying concentrations of CD123xCD3 or BCMAxCD3 were added to cultures for 48 hours. The percentage of dead CFSE^+^ cells was quantitated by flow cytometry. (A) Table showing a summary of the EC_50_ values of CD123xCD3 and BCMAxCD3 to mediate cytotoxicty of AML cell line KG-1 and MM cell line H929, respectively. (B) Similar experimental setup to A but here increasing amounts of HS-5 cells were added to KG-1-T cell cultures. (C) Ability of CD123xCD3 to mediate cytotoxicity of AML cell lines OCI-AML5 and MOLM-13, in the absence or presence of stroma was assesed. (D) Ability of BCMAxCD3 to mediate cytotoxicity of MM cell lines RPMI-8226 and MM.1S, in the absence or presence of stroma was assessed.

**Figure 3. Presence of stromal cells dampens T cell activation and proliferation**

(A) Human T cells were cultured with CFSE labelled MM cell line H929 at a 2:1 ratio in the presence or absence of stromal cells (HS-5, HS-27a, primary MSC or CD105^+^ endothelial cells). Varying concentrations of BCMAxCD3 were added to cultures for 48 hours. Geometric mean fluorescence intensities of T cell activation markers were quantified by flow cytometry at 48 hours post-treatment with bispecific antibodies. (B) Similar to A but here CFSE-labelled T cells were cultures with unlabelled tumor cell lines at a 2:1 ratio in the presence or absence of stromal cells (HS-5, primary MSC or CD105^+^ endothelial cells). FACS analyses showing overlay of CFSE dilution profiles (nullxCD3 control shown in gray while treatment group shown in black histograms), depicting T cell proliferation.

**Figure 4: Treatment with Bcl-2 inhibitor blocks expression of Bcl2.**

Immunoblotting analysis of expression of Bcl-2 in KG-1 cells cultured in the presence of HS5 stromal cell line and treated with or without Bcl-2i for 48 hours.

**Figure 5: Cell-cell contact and the VLA4 adhesion pathway plays a role in the stromal mediated suppression of cytotoxicity in MOLM-13 cells.**

(A) Human T cells were cultured with CFSE labelled MOLM-13 cells with or without HS-5 or primary MSC cells and were treated with varying concentrations of bispecific antibody for 48 hours. In these assays, stromal cells were either cultured together or separated from the tumor and T cells in a trans-well. The percentage of dead CFSE^+^ cells was quantitated by flow cytometry. (B) Similar to A but here cytotoxicity was assessed when all cells were cultured together and in the presence or absence of neutralizing antibodies to VLA4 or CXCR4.

**Figure 6: Treatment with VLA4 neutralizing antibody reduces phosphorylation of AKT and PI3K pathways.**

Immunoblotting analysis of expression of pAkt and pPI3K in KG-1 cells cultured in the presence of HS5 stromal cell line and treated with or without anti-VLA4 neutralizing antibody for 48 hours.

**Figure 7: Gating strategy for the AML primary patient samples**

Gating strategy for the ex vivo experiments conducted with primary AML patient samples. The corresponding isotype controls are shown on the side. (A) CD123^+^ blasts were identified by first gating on forward scatter (FSC) and side scatter (SSC) to isolate cells of interest. Live CD45^+^ cells were then gated on, after which CD38^+^ CD33^+^ blasts were gated on. Next, blasts expressing CD123 were quantified in the various conditions. (B) To quantify T cell expansion in the samples, we first gated on cells of interest with SSC/FSC and then identified live CD45^+^ cells. We then identified CD4^+^CD8^-^ and CD8^+^CD4^-^ T cells based on CD4 and CD8 staining.

**Figure 8: Gating strategy for the MM primary patient samples**

Gating strategy for the ex vivo experiments conducted with primary MM patient samples. The corresponding isotype controls are shown on the side. (A) CD138^+^ MM cells were identified by first gating on FSC/SSC to isolate cells of interest. Live CD138^+^ cells were then quantified in the various conditions. (B) To quantify the T cell activation in the samples, we first gated on lymphocytes with SSC/FSC and then identified live CD138^-^ cells. We then measured expression of CD25 on CD8^+^ T cells.
