# Supplementary material for: Blockade of VLA4 sensitizes leukemic and myeloma tumor cells to CD3 redirection in the bone marrow microenvironment
Source: Blood Cancer J. 2020 Jun 1;10(6):65. doi: 10.1038/s41408-020-0331-4 (PMC7264144; doi:10.1038/s41408-020-0331-4)
Supplement: Supplementary file 2 — Supplementary Methods [file 41408_2020_331_MOESM2_ESM.docx]

**Supplemental Methods**

**Cell lines**

KG1, H929, RPMI-8226, MM.1S, HS-5 and HS-27a cell lines were obtained from the American Tissue Culture Collection (Manassas, VA). MOLM‑13 and OCI-AML5 were obtained from Deutsche Sammlung von Mikroorganismen und Zellkulturen (DSMZ, Germany). Primary mesenchymal stem cells cryopreserved from normal human donors were purchased from Lonza (Basel, Switzerland) and CD105^+^ bone marrow endothelial cells were purchased from All Cells (Alameda, California). IncuCyte® NucLight Green or NucLight Red Lentivirus Reagent (EF1a, Puro) was purchased from Essen Bioscience (Ann Arbor, Michigan) and was used according to manufacturer’s instructions to generate HS-5-NucLight Green and OCI-AML5-NucLight Red cells. Puromycin treatment was used to select fluorescent positive cell lines. While the cell lines were not authenticated recently, they tested negative for mycoplasma contamination.

Binding assay with bispecific antibodies

All tumor cells were centrifuged, washed twice with Dulbecco’s phosphate-buffered saline (DPBS) and 1×10^4^ cells were added to the center of each well of a 96 well U bottom plate along with fragment crystallizable (Fc) block (human IgG1 fragment) which was added at 2 mg/mL for 10 minutes. Serially diluted bispecific antibodies were added to the appropriate wells. Plates were incubated in the dark at 37°C with 5% CO2 for 4 hours. The cells were then washed with DPBS and binding of the bispecific antibody was detected by staining with mouse anti-human IgG4 (Southern Biotech, clone HP6025, catalog# 9200-09) and LIVE/DEAD (L/D; Invitrogen, catalog# L34976) for 30 minutes. Finally, cells were washed, resuspended in stain buffer, and analyzed on the FACSCanto II flow cytometer (BD Biosciences). Geometric mean fluorescence intensity (gMFI) was plotted in Prism version 7 (GraphPad). The X axis was log transformed and a 4 parameter non-linear curve fit was applied.

***In vitro* cytotoxicity assays with cell lines**

Tumor cell lines (KG1, MOLM‑13, OCI-AML5, H929, RPMI-8226 and MM.1S) were counted and washed with DPBS before incubation with carboxyfluorescein succinimidyl ester (CFSE; resuspended in 150 µL dimethyl sulfoxide and diluted 1:10,000) at 1×10^7^ cells/mL of CFSE for 8 minutes at RT. Staining was quenched with HI FBS. Cells were washed in complete medium before resuspension at 2×10^5^ cells/mL in complete medium containing 1 mg/mL human IgG_1_ fragment, then incubated for 15 minutes. The purified frozen T cells (obtained from BioIVT (Westport, New York)) were thawed and resuspended at 1×10^6^ cells/mL. The T cells were isolated from whole blood by using Ficoll gradient (to isolate mononuclear cells) and negative selection post incubation at room temperature with an antibody cocktail (CD16, CD19, CD36, CD56 and CD66b) to remove the ‘unwanted’ cells. The stroma cell lines (HS-5 and HS-27a) were harvested, washed, counted and resuspended at 4×10^5^ cells/mL. In the case of primary mesenchymal stromal cells (MSC) and CD105^+^ endothelial cells, frozen aliquots sourced from Lonza and All cells respectively, were thawed and resuspended at 4×10^5^ cells/mL. Finally, 50 µL of purified T cells, 50 µL of stromal cells and 100 µL of labeled tumor cells were combined in each well of a 96‑well U bottom plate with 0.5 mg/mL human IgG_1_ fragment. 24 hours later, the test antibodies were added to the wells. The antibodies were diluted to a final starting concentration of 133 nM in DPBS or complete medium. The antibodies were further diluted 3-fold and added to appropriate wells. All plates were incubated at 37°C with 5% CO_2_ for 48 hours post addition of antibodies. The cells were then washed with DPBS and stained for various markers before analyzing on the flow cytometers.

For the proliferation experiments, the in vitro assays were performed as detailed above except that here T cells were labelled with the CFSE dye prior to co-culture, thus allowing assessment of proliferation by monitoring CFSE 96 hours post addition of the bispecific antibodies.

For the transwell related experiments, the assay was performed in 96 well U bottom plates with or without 0.4 µM transwell inserts (HTS TRANSWL96, Corning). The stromal cells were either combined with T and tumor cells or separated from the T and tumor cells by seeding on the transwell insert.

For the IncuCyte^®^ related experiments, red fluorescent OCI-AML5 cells were used (OCI-AML5-NucLight Red) and green HS-5 (HS-5-NucLight Green). Tumor, stroma and T cells were washed and combined in phenol-red-free RPMI / 10% HI FBS for these assays. Images of red and green objects (indicating red OCI-AML5 and green HS-5) per well were recorded by the IncuCyte^®^ Zoom every 6 hours over a time course of 120 hours.

For blocking experiments, the following inhibitors and neutralizing antibodies were used: Bcl-2 inhibitor (HA14-1), anti-human CXCR4 (12G5) and anti-human ITGA4/VLA4 (2B4) antibody were purchased from R&D systems (Minneapolis, Minnesota).

***Ex vivo* cytotoxicity assays with primary AML and MM patient samples**

30,000 or 600,000 HS-5 cells were plated per well of a 6 well plate overnight. Next morning, media was carefully removed before replacing with 3x10^6^ primary AML or MM PBMCs and BMMC, respectively in αMEM+10% FBS with 0.5 mg/mL human IgG_1_ fragment. Next, CD123xCD3, BCMAxCD3 or nullxCD3 bispecific antibodies (1 µg/ml) with or without anti-VLA4 antibody (5 µg/ml) were added. 72 hours later, depletion of CD123^+^ blasts or CD138^+^ MM plasma cells was monitored via flow cytometry. Additionally, expansion of CD8 T cells as well as their activation status (upregulation of CD25) were assessed.

**Flow cytometry and antibody reagents**

Antibodies for FACS included the following anti-human antibodies: CD278/ICOS (DX-29), CD4 (SK3), Granzyme B (GB11) (purchased from BD Biosciences), CD8 (RPA-T8), 41BB/CD137 (4B4-1), CD25 (BC96), Perforin (dG9), Tbet (4b10), PD-1/CD279 (EH12.2H7), TIM3 (F38-2E2), CD33 (WM53), CD38 (HIT2), CD123 (6H6), CD138 (MI15) (purchased from BioLegend), LAG3 (3DS223H) (purchased from eBiosciences) and LIVE/DEAD Near-IR (Life Technologies).

For FACS analysis, the plates were centrifuged at 1,500 rpm for 5 minutes. The cells were then washed with DPBS and stained for T cell activation markers and for cytotoxicity for 30 minutes. Finally, cells were washed and resuspended in stain buffer. For intracellular staining, cells were fixed and permeabilised using the IC Staining kit (eBiosciences) according to manufacturer’s instructions with minor modifications (washing four times with permeabilization buffer before incubation with intracellular cytokine antibody).

Data was acquired on a FACSCanto II (BD Biosciences) or LSRFortessa ((BD Biosciences). Tumor cell death was assessed by gating on forward-scatter (FSC) and side-scatter (SSC) to identify cell populations, then CFSE^+^ tumor events, and finally LIVE/DEAD Near-IR to assess tumor cell cytotoxicity. The L/D+ gate was drawn after comparing to the PBS treated and isotype controls. These controls also help account for errors related to non-specific binding of antibodies or spillover effects. T‑cell activation was assessed by gating on FSC and SSC to identify cell populations, CFSE^-^ events, live cells, then looking for positive staining for several markers. The percentage of either dead tumor cells was graphed using Prism 8 and analyzed with a 4‑parameter non-linear regression curve fit. For the T cell activation markers, the geometric mean fluorescent intensities of various markers were analyzed via FlowJo software and were graphed using Prism 8.

**Immunoblotting and antibody reagents**

Automatic western blots were performed using a Wes automated system (ProteinSimple, California, USA) according to manufacturer’s instructions. Samples were mixed with a 5x sample buffer containing SDS, DTT and fluorescent molecular weight standards and heated at 95 °C for 5 min and then, loaded onto a plate prefilled with stacking and separation matrices, along with blocking and wash buffers, antibody solutions and detection reagents. Default settings were used for the analysis. The following anti-human antibodies purchased from Cell Signaling Technology (Danvers, MA) were used to detect proteins: Bcl-2 (#2872), Phospho-p38 MAPK (Thr180/Tyr182) (D3F9) XP® Rabbit mAb (#4511), Phospho-Akt (Ser473) (D9E) XP® Rabbit mAb (#4060) and β-Actin (D6A8) Rabbit mAb (#8457).

**Animals**

Female NSG (NOD scid gamma or NOD.Cg-Prkdc^scid^ Il2rg^tm1Wjl^/SzJ) mice (The Jackson Laboratory, Bar Harbor, ME) were utilized when they were approximately 6-8 weeks of age and weighed 20 g. All animals were allowed to acclimate and recover from any shipping-related stress for a minimum of 5 days prior to experimental use. Reverse osmosis (RO) chlorinated water and irradiated food (Laboratory Autoclavable Rodent Diet 5010, Lab Diet) were provided ad libitum, and the animals were maintained on a 12 hour light and dark cycle. Cages, bedding and water bottles were autoclaved before use and changed weekly. All experiments were carried out in accordance with The Guide for the Care and Use of Laboratory Animals and were approved by the Institutional Animal Care and Use Committee of Janssen R&D, Spring House, PA.
