# Supplementary figures and images for: Blockade of VLA4 sensitizes leukemic and myeloma tumor cells to CD3 redirection in the bone marrow microenvironment
Source: Blood Cancer J. 2020 Jun 1;10(6):65. doi: 10.1038/s41408-020-0331-4 (PMC7264144; doi:10.1038/s41408-020-0331-4)

A

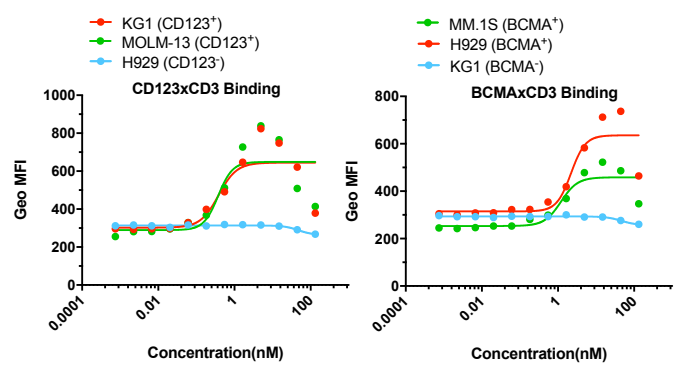

B

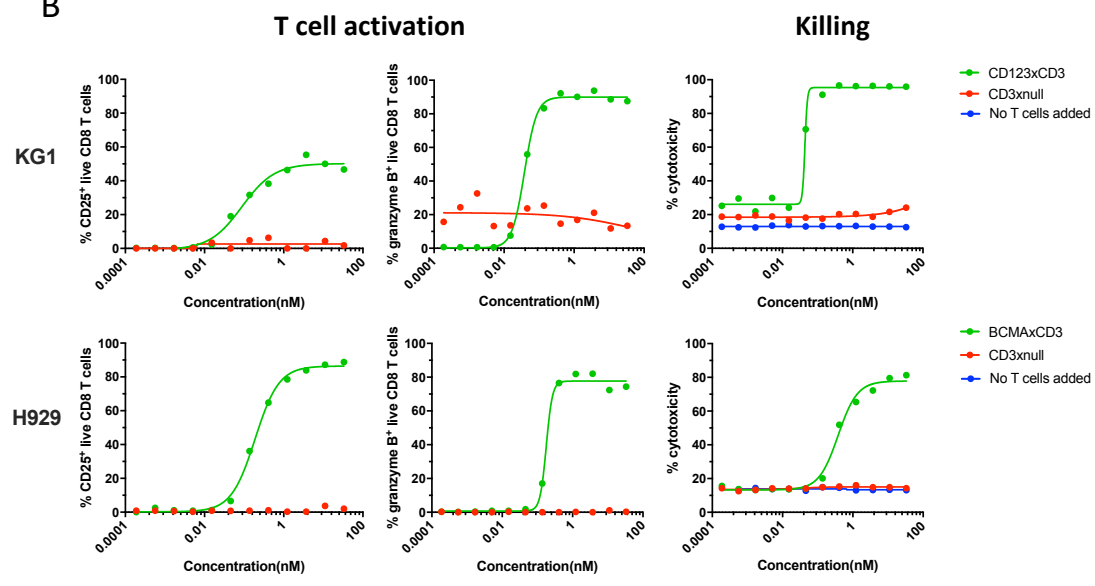

Supplementary Figure 1

Supplement: Supplementary file 3 — Supplemental Figure 1 [file 41408_2020_331_MOESM3_ESM.pdf]

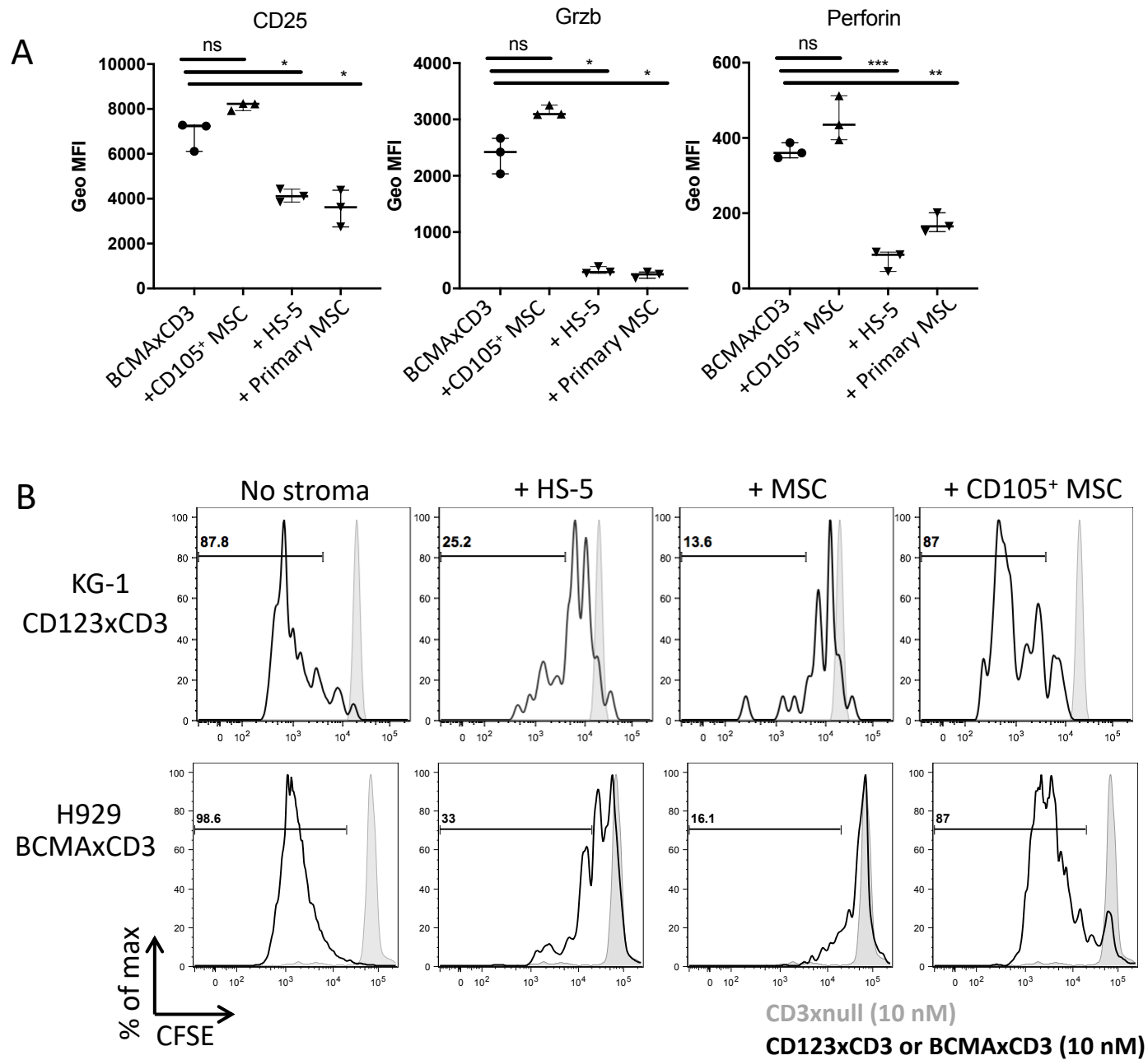

**Supplementary Figure 3**

Supplement: Supplementary file 5 — Supplemental Figure 3 [file 41408_2020_331_MOESM5_ESM.pdf]

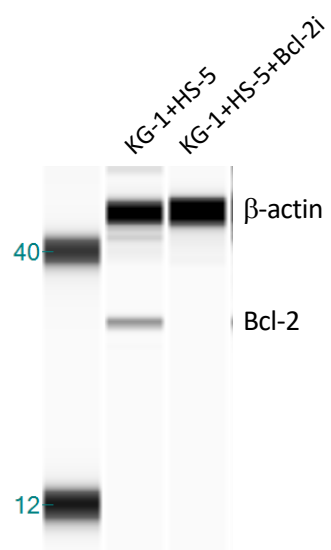

**Supplementary Figure 4**

Supplement: Supplementary file 6 — Supplemental Figure 4 [file 41408_2020_331_MOESM6_ESM.pdf]

A

MOLM-13, CD123xCD3

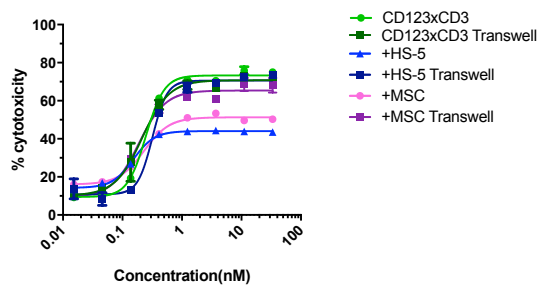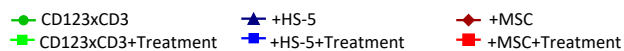

B

MOLM-13, CD123xCD3

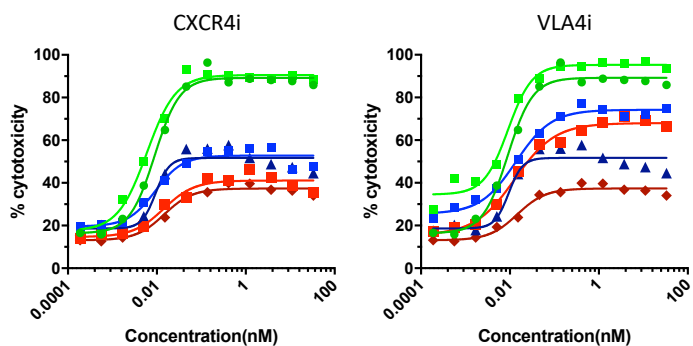

Supplementary Figure 5

Supplement: Supplementary file 7 — Supplemental Figure 5 [file 41408_2020_331_MOESM7_ESM.pdf]

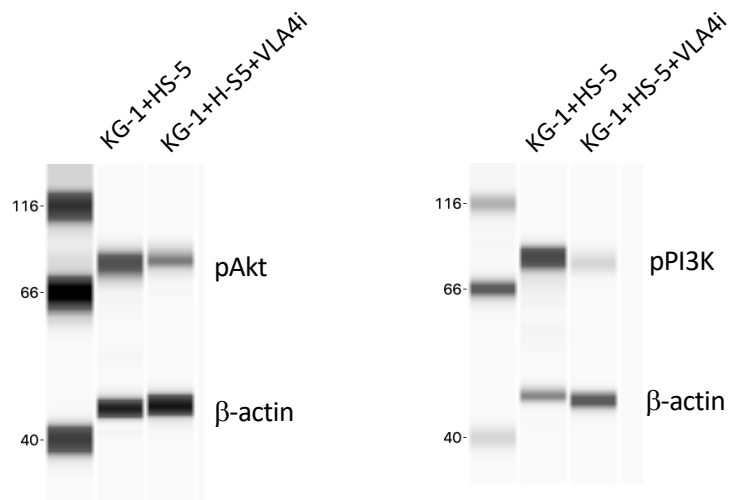

**Supplementary Figure 6**

Supplement: Supplementary file 8 — Supplemental Figure 6 [file 41408_2020_331_MOESM8_ESM.pdf]

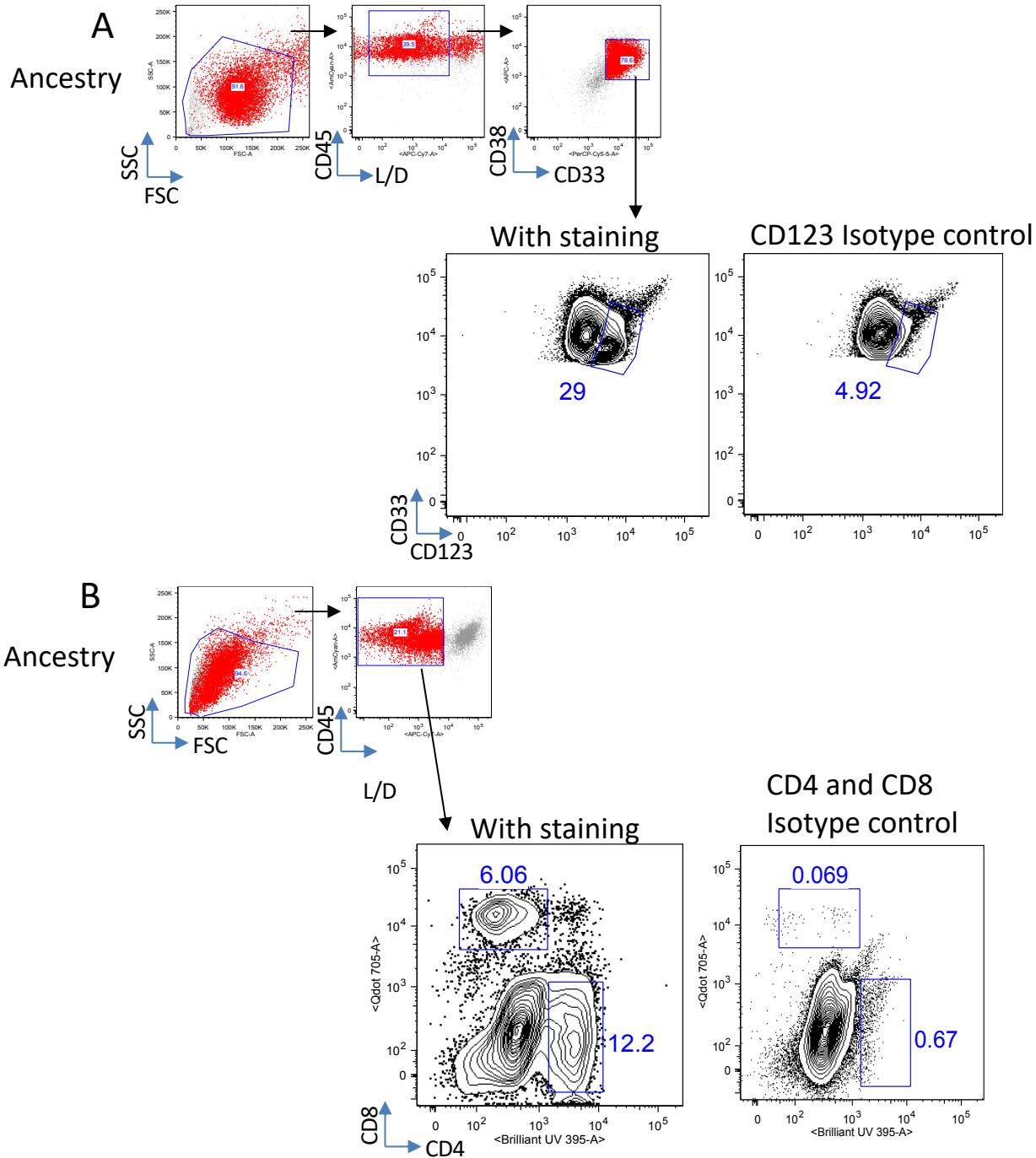

**Supplementary Figure 7**

Supplement: Supplementary file 9 — Supplemental Figure 7 [file 41408_2020_331_MOESM9_ESM.pdf]

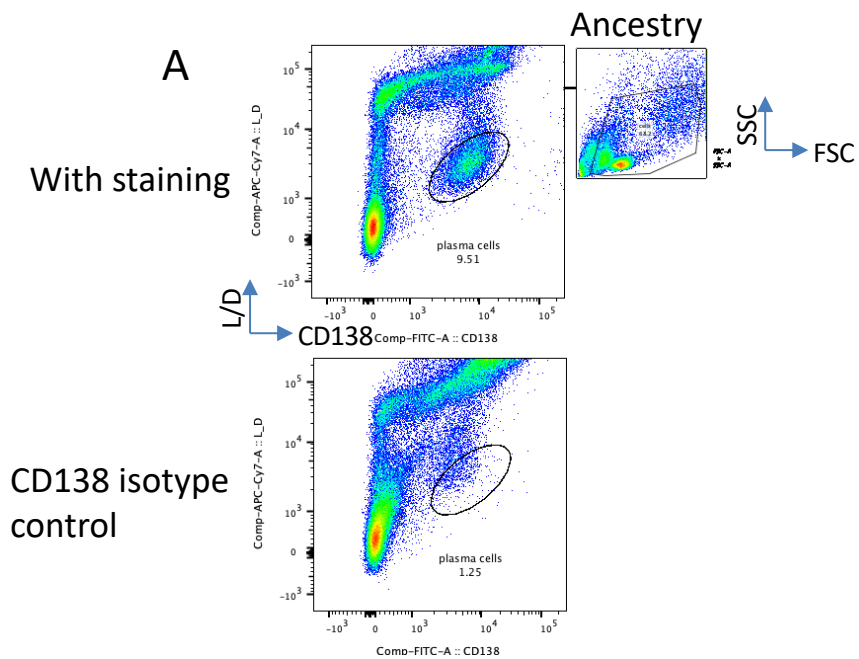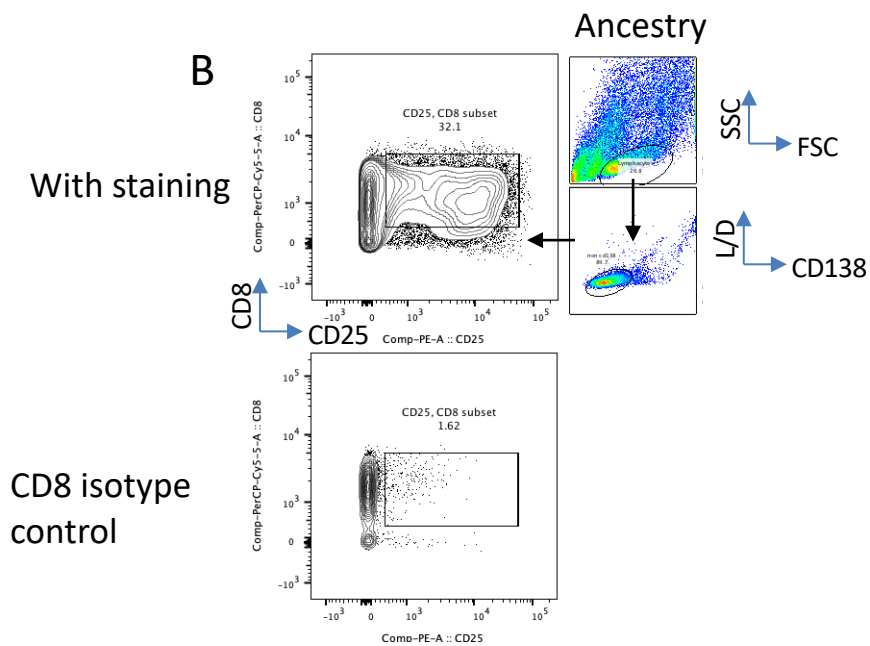

**Supplementary Figure 8**

Supplement: Supplementary file 10 — Supplemental Figure 8 [file 41408_2020_331_MOESM10_ESM.pdf]
