# Supplementary material for: Blockade of VLA4 sensitizes leukemic and myeloma tumor cells to CD3 redirection in the bone marrow microenvironment
Source: Blood Cancer J. 2020 Jun 1;10(6):65. doi: 10.1038/s41408-020-0331-4 (PMC7264144; doi:10.1038/s41408-020-0331-4)
Supplement: Supplementary file 4 — Supplemental Figure 2 [file 41408_2020_331_MOESM4_ESM.pdf]

A

Sample	KG-1 Cytotoxicity EC_{50} (nM)	H929 Cytotoxicity EC_{50} (nM)
	CD123xCD3	BCMAxCD3
Bispecific Ab	0.005	0.23
Bispecific Ab+HS-5	0.02	0.70
Bispecific Ab+HS-27a	0.03	0.30
Bispecific Ab+MSC	0.03	1.23
Bispecific Ab+CD105^{+} endothelial cells	0.008	0.17
	N = 3 T cell donors	

B

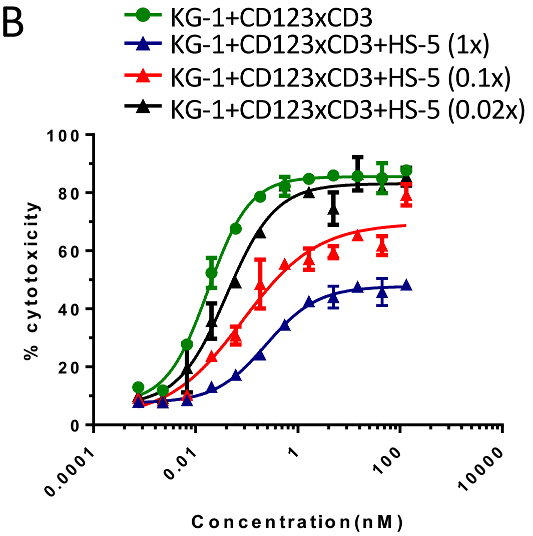

C

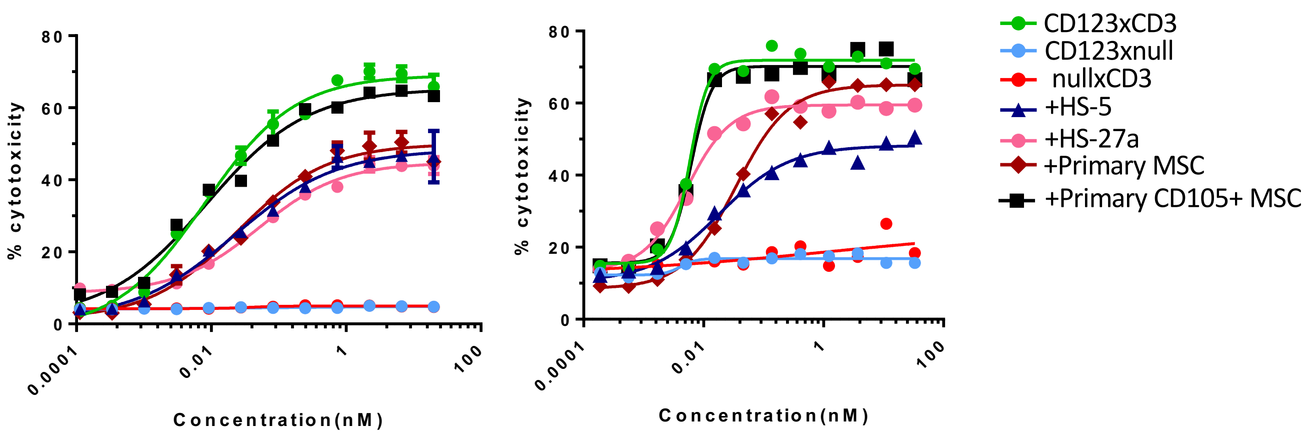

D

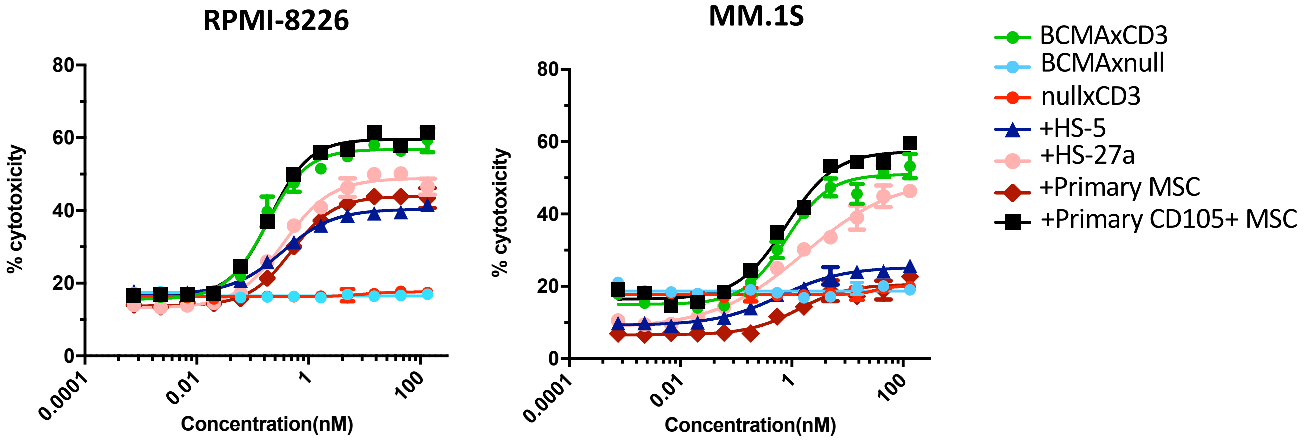

Supplementary Figure 2
